# Machine learning-based exploration of the associations between multiple minerals' intake and thyroid dysfunction: data from the National Health and Nutrition Examination Survey

**DOI:** 10.3389/fnut.2025.1522232

**Published:** 2025-03-26

**Authors:** Shaojie Liu, Weibin Huang, Yaming Lin, Yifei Wang, Hongjin Li, Xiaojuan Chen, Yijia Zou, Bo Chen, Baochang He, Zhiping Yang, Jing Fan

**Affiliations:** ^1^The First Affiliated Hospital of Xiamen University, School of Medicine, Xiamen University, Xiamen, China; ^2^Key Laboratory of Public Health Safety of Ministry of Education, School of Public Health, Fudan University, Shanghai, China; ^3^Department of Neurology, The First Affiliated Hospital of Fujian Medical University, Fuzhou, China; ^4^Institute for Infectious Disease Control and Prevention, Fujian Provincial Center for Disease Control and Prevention, Fuzhou, China; ^5^School of Public Health, Fujian Medical University, Fuzhou, China

**Keywords:** minerals, hyperthyroidism, hypothyroidism, machine learning model, dietary health

## Abstract

**Objectives:**

The associations between various minerals' intake and thyroid dysfunction (TD), including hyperthyroidism and hypothyroidism, are still inconclusive, which may be attributed to the potential synergistic effects among various minerals.

**Methods:**

The data were obtained from the National Health and Nutrition Examination Survey (NHANES) 2001–2002 and 2007–2012 databases. Dietary interviews were conducted to collect the consumption of multiple minerals. Blood samples were collected to measure concentrations of free triiodothyronine, free thyroxine, and thyroid-stimulating hormone. A total of 7,779 participants with aged over 20 years were effectively enrolled in this study and categorized into hyperthyroidism or hypothyroidism groups. Weighted multivariate logistic regression model along with three machine learning models WQS, qg-comp, and BKMR were employed to investigate the individual and joint effect of multiple minerals' consumption on TD.

**Results:**

Among 7,779 subjects, 134 participants were diagnosed as hyperthyroidism and 184 participants were diagnosed as hypothyroidism, with prevalence of 1.6 and 2.4%, respectively. The results from logistic regression model showed that the higher the intakes of calcium, magnesium and potassium, the lower the prevalence of hyperthyroidism, with OR values of 0.591, 0.472, and 0.436, respectively (all *P* < 0.05); while the higher the intake of iodine, the higher the prevalence of hyperthyroidism, with OR and 95%CI values of 1.262 (1.028, 1.550). Three machine learning models were employed to evaluate the joint effect of nine minerals' consumption on TD, revealing a negative correlation with both hyperthyroidism and hypothyroidism. Of them, the potential minerals associated with TD were calcium, zinc, copper, and magnesium.

**Conclusion:**

In short, the maintenance of a well-balanced consumption of multiple minerals is considered crucial in the prevention and treatment of TD, and the intakes of various minerals exhibit varying degrees of association with TD.

## 1 Introduction

Thyroid dysfunction (TD) including hypothyroidism and hyperthyroidism is a great common endocrine disorder worldwide, whose characteristics is of the over-expression or low-expression of blood thyroxine (T4), triiodothyronine (T3), and thyroid stimulating hormone (TSH). At present, the prevalence and incidence of TD reported by epidemiological studies are increasing gradually and different significantly in various countries and regions (1). Madariaga et al. conducted a meta-analysis in European population and found that the prevalence of TD was of 3.05 and 0.75% for hypothyroidism and hyperthyroidism, respectively (2). Another U.S. study revealed that 4.6% of the population had hypothyroidism and 1.3% had hyperthyroidism by analyzing the data of National Health and Nutrition Examination Survey (NHANES) III (3). In China, the prevalence is high to 13.95% for hypothyroidism and 1.22% for hyperthyroidism (4). It exhibits that TD has emerged as a global public health issue, necessitating an exploration of the factors influencing its progression.

Multiple factors participated in the occurrence and development of TD, such as age, gender, educational attainment, cigarette smoking, alcohol drinking, heredity, and diet (5–8). Diet especially for the minerals' consumption may be regarded as one of the most important factors of association with TD (9). Epidemiological studies found that people with chronic mild to moderate iodine deficiency had a higher incidence of hyperthyroidism than those with normal or excessive iodine intake (10). While increased iodine intake may lead to an increased risk of subclinical hypothyroidism (11). Pedersen et al. conducted a case-control study and revealed that compared to control group, there was a significantly lower serum selenium level for patients with newly diagnosed Graves' disease and autoimmune overt hypothyroidism (12). Another cross-sectional study showed a comparable prevalence of hyperthyroidism in both counties, irrespective of selenium intake (13). Meanwhile, low zinc concentrations were reportedly associated with both hypothyroidism and hyperthyroidism (14). The impact of manganese on the thyroid remains poorly comprehended. One study clarified that high serum manganese concentrations would reduce free thyroxine and triiodothyronine levels, leading to hypothyroidism (15). Above studies suggest that the relationships between minerals' consumption and TD is still unclear and has the contradictory results, which can be ascribed to the synergistic interaction among various minerals.

Diet comprising a variety of food items needs to be considered as a whole, involving the co-consumption of multiple minerals. There may be synergistic interactions among various minerals for maintaining the normal thyroid function. For example, the interaction between iodine and selenium plays a crucial role in thyroid metabolism. Excessive intake of selenium exacerbates the consequences of iodine deficiency, whereas an appropriate supply of selenium can mitigate the adverse effects of excessive iodine on thyroid function and prevent inflammation, fibrosis, and destruction (16). However, the majority of studies failed to account for the synergistic interactions of multiple minerals' intake and instead focused solely on the relationships between individual or paired minerals' intake and TD using linear or logistic regression models (12, 17). This may be associated with the limitation of statistical methods. Multiple minerals' intake is inappropriate to be simultaneously put into the linear or logistic regression model due to collinearity question. Thus, when evaluating the synergistic interactions of multiple minerals' intake, traditional models like multiple linear regression and multivariate logistic regression may not provide accurate results.

In the field of nutrition, machine learning approaches, involving weighted quantile sum (WQS), quantile g-computation (qg-comp), bayesian kernel machine regression (BKMR) and others can address the multicollinearity of multiple variables through sophisticated internal algorithms. These methods are increasingly being adopted to supplement traditional statistical techniques, with the goal of generating more scientifically robust conclusions. Growing number of researchers have started applying machine learning techniques to explore the complex relationships between the co-consumption of various nutrients and health outcomes (18, 19). However, there is a scarcity of studies examining the relationship between the combined intake of multiple minerals and TD. Based on current scientific evidence, we propose that the concurrent consumption of multiple minerals may be protectively associated with the prevalence of TD. Thus, it is imperative to employ machine learning methods to delve the deep association between simultaneous consumption of multiple minerals and TD.

In this study, totals of 7,779 eligible participants were enrolled from National Health and Nutrition Examination Survey (NHANES) database. The novel machine learning methods were used to unveil the associations between consumption of multiple minerals and TD, and further elucidate their joint effect and the individual contribution. To our best knowledge, this study is the first time to explore the combined interaction between the consumption of multiple minerals and TD by the emerging machine learning methods in U.S. adults.

## 2 Materials and methods

### 2.1 Study population

**Figure 1:**
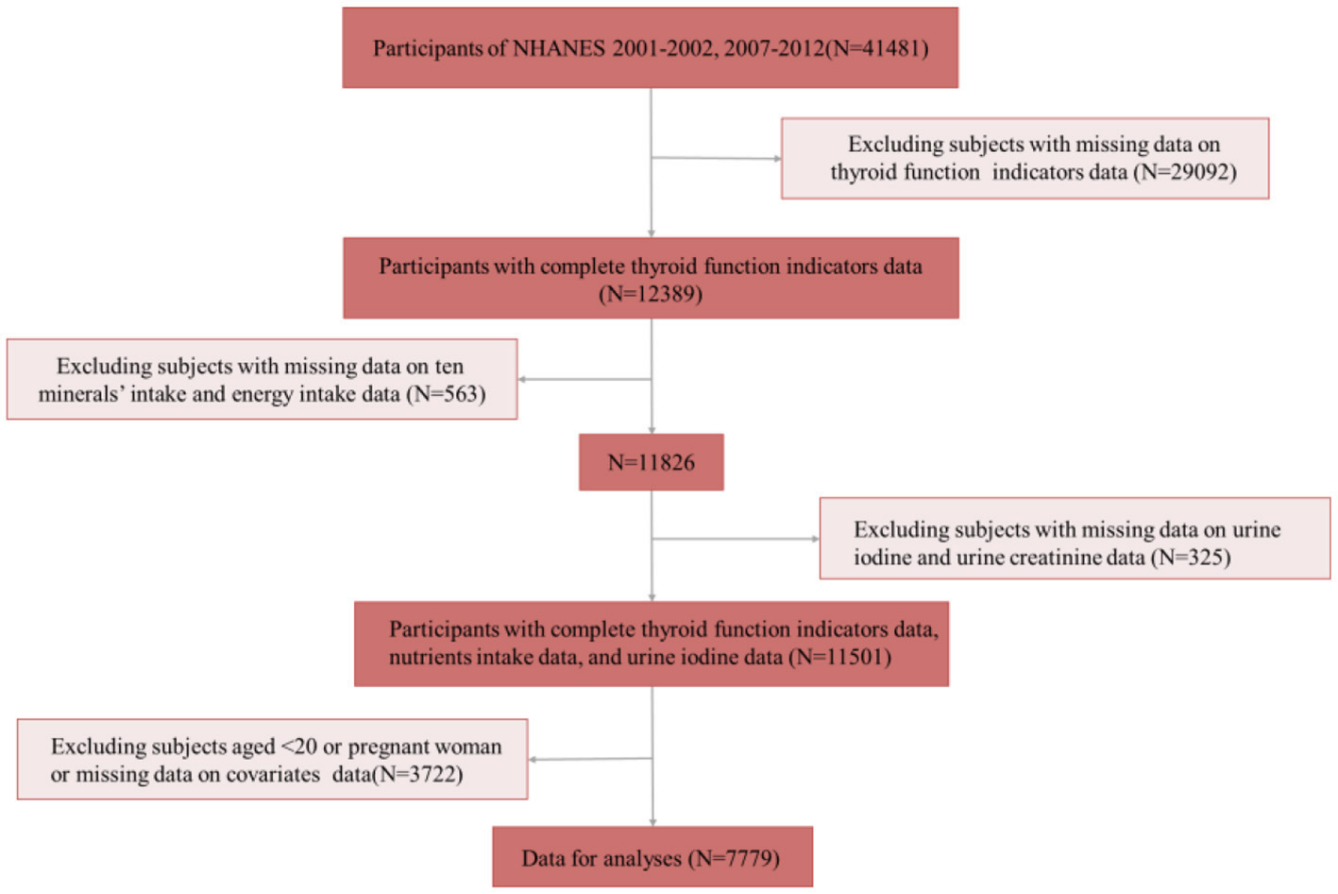
Flow chart for study participants selection.

NHANES used a stratified multistage sampling design to conduct a nationwide survey that investigated detailed information about the health, nutrition, and lifestyle of U.S. residents. All surveys were reviewed by the Ethics Committee and received informed consent from the participants. All details are available on the NHANES official website and data are supported for local download (https://www.cdc.gov/nchs/nhanes/index.htm). Four cycles of NHANES (2001–2002, 2007–2008, 2009–2010, and 2011–2012) were included in this study. A total of 41,481 participants were selected from the NHANES datasets spanning 2001–2002 and 2007–2012. Participants with missing data on thyroid function (*N* = 29,092), mineral intake and energy intake (*N* = 563), urinary iodine and creatinine levels (*N* = 325), as well as those lacking covariate data, aged under 20 years, or who were pregnant (*N* = 3,722) were excluded. Consequently, a final sample of 7,779 participants was included in this study. The flow chart is shown in Figure 1.

### 2.2 Assessment of multiple minerals' intake

Minerals' intake data were directly available in NHANES. It is worth noting that only one 24-h dietary recall was conducted in the 2001–2002 wave, whereas two 24-h dietary recalls were done in the other three waves included in the study. Therefore, when processing data on average daily mineral intake, for participants with only one day of 24-h dietary data, we used the minerals consumed during that day as their representative intake level. For participants with 2 days of 24-h dietary data, we used the average of the minerals consumed on both days as their representative intake level. A total of nine minerals were ultimately included in the study: calcium, iron, zinc, selenium, magnesium, phosphorus, potassium, copper, and iodine. However, NHANES does not provide the data of iodine intake. Considering that iodine is an important influence on thyroid function, the current study included creatinine-corrected urinary iodine as the participants' iodine intake level. Urinary iodine testing methods can be found on the official website.

### 2.3 Definition of hyperthyroidism and hypothyroidism

NHANES contains blood test data for thyroid function indicators. The indicators utilized in this study included free thyroxine (FT4), free triiodothyronine (FT3) and thyroid stimulating hormone (TSH). Serum FT4, FT3, and TSH levels were measured by ELISA kit, and light generated by the reaction was measured with a luminometer. The light production was positively or negatively proportional to the concentrations of serum FT4, FT3, and TSH in the sample. The amount of analyte was determined from a stored, multi-point calibration curve. Reference ranges for these three markers are listed in the laboratory procedures manual, with TSH being 0.30–5.60 μIU/mL, FT3 being 2.50–3.90 pg/mL, and FT4 being 0.60–1.60 ng/dL (20). With reference to previous categorizations in the literature, we categorized the thyroid function indices of the participants as less than the reference range, within the reference range and beyond the reference range. Accordingly, four categories were distinguished among the participants: clinical hyperthyroidism, subclinical hyperthyroidism, clinical hypothyroidism and subclinical hypothyroidism. Clinical hyperthyroidism was defined as TSH lower than the reference range and FT3 or FT4 higher than the reference range; subclinical hyperthyroidism was defined as TSH lower than the reference range and FT3 and FT4 within the reference range; clinical hypothyroidism was defined as TSH higher than the reference range and FT3 or FT4 lower than the reference range; and subclinical hypothyroidism was defined as TSH higher than the reference range and FT3 or FT4 within the reference range (20). We combined clinical and subclinical hyperthyroidism and uniformly declared it as hyperthyroidism. Clinical and subclinical hypothyroidism were combined and uniformly declared as hypothyroidism.

### 2.4 Covariates

Similar to the previous NHANES study (20, 21), the following confounders were selected as covariates: i) Categorical variables: sex (male, female), age (under 35, 35–50, 50–65, 65 and over), race/ethnicity (Mexican American, Other Hispanic, non-Hispanic White, non-Hispanic Black, Other Race-Including Multi-Racial), energy intake (divided into four categories by quartile), marital status (married, widowed, divorced, separated, never married, living with partner), educational attainment [ < 9th grade, 9th−11th grade (includes 12th grade with no diploma), high school Grad/GED or equivalent, some college or AA degree, college graduate or above], drinking status (no drinking, light drinking, moderate drinking, heavy drinking, and missing categories); and (ii) continuous variables: blood cotinine concentration (as an indicator of smoke exposure level), poverty-to-income ratio (PIR), body mass index (BMI).

### 2.5 Statistical analyses

Categorical variables were presented as frequencies and percentages, and continuous variables as means (standard deviations). Certain variables in mineral intake fail to align precisely with the normal distribution by the Shapiro-Wilk test, including iodine, copper, potassium, phosphorus and magnesium; therefore, the intake of all minerals was log-transformed to achieve normality and consistency, facilitating the joint effect analysis using machine learning methods. Sex, age, race, education, poverty-to-income ratio, energy intake, BMI, marriage, alcohol consumption, serum cotinine, and alcohol consumption were included as covariates to adjust all models for decreasing the potential biases. For preliminary analyses, we set hyperthyroidism and hypothyroidism as dichotomous variables (1 for event and 0 for non-event) to perform weighted multivariate logistic regression. In order to avoid multicollinearity between the independent variables, instead of including all minerals in the same model, we included each mineral in its own separate model and output their ORs and 95% confidence intervals. Compared to traditional statistical methods, machine learning models offer several advantages. They can be applied to a wide range of data types and tasks, effectively handle noise and outliers in data, reduce reliance on parameter selection, and capture complex non-linear relationships. Additionally, machine learning models are better suited for handling multivariate and high-dimensional data, thereby enhancing the reliability of results and improving analysis efficiency. Thus, we further evaluated the combined effects of nine minerals on hyperthyroidism and hypothyroidism using three state-of-the-art statistical methods, namely, WQS, qg-comp, and BKMR, and identified minerals with higher weighted effects in the joint effect.

### 2.6 Introduction of machine learning methods

WQS was utilized to quantify both the mixing effect and the degree to which individual components were characterized within it (22). It constructs a weighted index of mixed effects and includes penalized weight estimates in the built-in function to identify the individual weights of the components (22). We fitted the model using quarticied independent variables and set the ratio of training and validation sets to 4:6, performing 10,000 iterations. Mineral intake with an estimated weight >0.111 (1/9) was considered to have a significant effect on the joint effect. However, there is an obvious limitation to the WQS in that the direction of the total effect needs to be constrained in advance as either positive or negative, which restricts the accuracy of parameter estimates with effects in different directions (22). Qg-comp has similar characteristics to WQS in that it can estimate mixed effects and output individual weights. Wonderfully, compared to WQS, qgcomp does not constrain a consistent direction, which makes the results more reliable. The qgcomp.boot function was used to estimate the combined effect of the nine minerals on hyperthyroidism and hypothyroidism. The qgcomp.noboot function was used to estimate the weights of each mineral in a positive or negative direction. Estimated weights above 0.05 were considered to have a higher effect in the total effect (19). The BKMR processes the model using probabilistic regression rather than logistic regression, with an embedded set of functions to address the issue of mixed exposures in relation to outcomes (23). The high-dimensional functions make it feasible to estimate the joint effects of mixed exposures as well as the interactions between exposures (23). Different machine learning methods possess distinct advantages in statistical analysis. In the field of nutrition, three primary machine learning methods—WQS, qg-comp, and BKMR—are commonly employed to investigate the associations of combined nutrients' intake with health outcomes (18, 19). Therefore, we selected these three common machine learning approaches to examine the relationships between multiple minerals' consumption and TD in this study.

## 3 Results

As shown in Table 1, totals of 7,779 study subjects were eligibly enrolled in this study, including 3,963 males and 3,816 females. The prevalence of hypothyroidism and hyperthyroidism was 2.4% (2.0%, 2.7%) and 1.6% (1.3%, 1.9%), respectively. The means of BMI, energy intake, and urine iodide were 28.57 kg/m^2^, 2148.28 kcal, and 5.93 μg/L Cre. The majority of study subjects were of 35–49 years old (30.6%), non-Hispanic White (72.2%), married (56.8%), and moderate alcohol drinkers (26.5%).

**Table 1:** The demographic characteristics of study subjects (*n* = 7,779).

**Demographic characteristics**	***n* (weight%)/Mean (SD)**
**Age (years)**	
20–34	1,872 (27.1)
35–49	2,007 (30.6)
50–64	2,031 (26.2)
65-	1,869 (16.1)
**Sex**	
Male	3,963 (49.4)
Female	3,816 (50.6)
**BMI (kg/m** ^2^ **)**	28.57 (0.1)
**Race/ethnicity**	
Mexican American	1,276 (7.6)
Other Hispanic	722 (4.8)
Non-Hispanic White	3,843 (72.2)
Non-Hispanic Black	1,492 (9.8)
Other Race-Including Multi-Racial	446 (5.6)
**PIR**	
Below poverty (< 1.0)	6,249 (86.5)
Above (≥1.0)	1,530 (13.5)
**Education level**	
Less than 9th grade	866 (5.6)
9–11th grade	1,289 (12.4)
High School graduate/GED	1,808 (23.8)
Some college or associate degree	2,170 (30.5)
College graduate or above	1,646 (27.7)
**Marital status**	
Married	4,156 (56.8)
Widowed	648 (5.7)
Divorced	822 (9.9)
Separated	259 (2.4)
Never married	1,315 (17.6)
Living with partner	579 (7.5)
**Alcohol consumption**	
Never	2,133 (23.2)
Mild	1,874 (25.7)
Moderate	1,775 (26.5)
Heavy	1,082 (14.7)
Unknown	915 (9.9)
**Energy intake (kcal)**	2148.28 (14.1)
**Urine Iodide (μg/L Cre)**	5.93 (3.2)
**Serum cotinine (ng/mL)**	62.3 (3.5)
**Hypothyroidism**	
Yes	184 (2.4)
No	7,595 (97.6)
**Hyperthyroidism**	
Yes	134 (1.6)
No	7,645 (98.4)

SD, standard deviation; BMI, body mass index; FIR, family income to poverty ratio; GED, general educational development.

The associations between each mineral intake and TD by multivariate logistic regression were shown in Table 2. After adjusting for co-variables, the results showed that the higher intake of calcium, magnesium, and potassium were associated with lower hyperthyroidism prevalence, with the OR (95% CI) values of 0.591 (0.370, 0.943), 0.472 (0.232, 0.960), and 0.436 (0.230, 0.829), respectively. The higher intake of iodide was associated with higher hyperthyroidism prevalence, with a OR (95% CI) value of 1.262 (1.028, 1.550). However, there was no significant association between each mineral intake and hypothyroidism found in multivariate logistic regression (all *P* > 0.05).

**Table 2:** The associations between each mineral intake and thyroid dysfunction by multivariate logistic regression.

**Minerals' intake**	**Hypothyroidism**		**Hyperthyroidism**	
	**OR (95%CI)**	***P*-value**	**OR (95%CI)**	***P*-value**
Calcium	0.639 (0.398, 1.025)	0.063	0.591 (0.370, 0.943)	0.028
Magnesium	0.673 (0.358, 1.265)	0.215	0.472 (0.232, 0.960)	0.038
Phosphorus	0.677 (0.300, 1.527)	0.341	0.576 (0.273, 1.215)	0.145
Iron	0.746 (0.447, 1.245)	0.257	0.816 (0.431, 1.545)	0.527
Zinc	0.915 (0.594, 1.411)	0.683	0.663 (0.381, 1.153)	0.143
Copper	0.885 (0.541, 1.449)	0.623	0.680 (0.316, 1.463)	0.319
Potassium	0.659 (0.388, 1.120)	0.121	0.436 (0.230, 0.829)	0.012
Selenium	0.859 (0.422, 1.747)	0.670	0.777 (0.430, 1.401)	0.395
Iodide	1.089 (0.840, 1.412)	0.512	1.262 (1.028, 1.550)	0.027

All models were adjusted by age, gender, energy intake, serum cotinine, BMI, PIR, marital status, alcohol consumption, educational level, and race/ethnicity.

We adopted the WQS and qg-comp models to explore the overall effect of multiple minerals on TD, as shown in Table 3. After adjusting for co-variables, the WQS model showed the overall effect of these nine minerals was negatively related with hyperthyroidism (estimate value: −0.350), with the difference being statistically significant (*P* = 0.05); the qg-comp model revealed the similar result (estimate value: −0.187) although there were no statistically significant (*P* > 0.05). Meanwhile, the overall effects of these nine minerals were negatively related with hypothyroidism, with the estimate values of −0.054 for WQS model and −0.113 for qg-comp model (*P* > 0.05).

**Table 3:** The overall effects of mixed minerals' intake on thyroid dysfunction obtained by WQS model and qg-comp model.

**Model**	**Outcomes**	**Estimate**	**Std. Error**	***P* value**
WQS	Hypothyroidism	−0.054	0.170	0.750
	Hyperthyroidism	−0.350	0.179	**0.050**
Qg-comp	Hypothyroidism	−0.113	0.141	0.425
	Hyperthyroidism	−0.187	0.164	0.252

All models were adjusted by age, gender, energy intake, serum cotinine, BMI, PIR, marital status, alcohol consumption, educational level, and race/ethnicity. WQS, weighted quantile sum. The bold value indicated that *P* value was less than or equal to 0.05.

**Figure 2:**
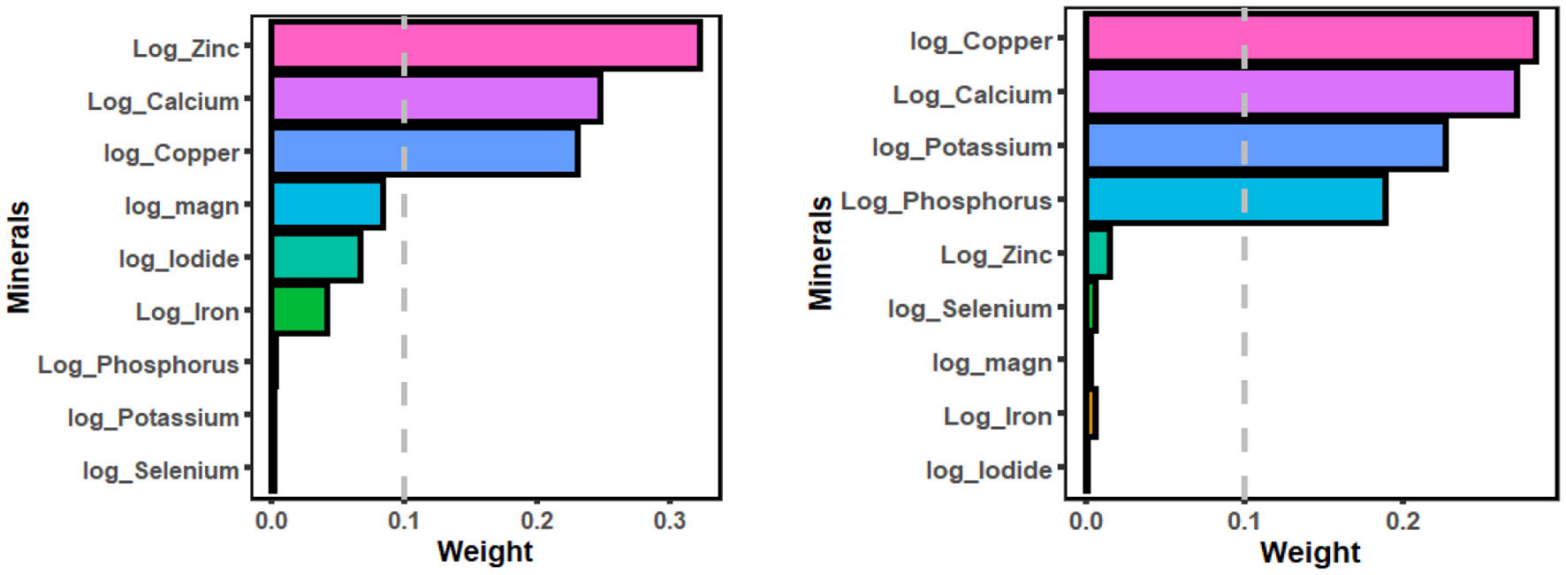
WQS model regression index weights for hypothyroidism (figure left) and hyperthyroidism (figure right). The WQS models were adjusted by age, gender, energy intake, serum cotinine, BMI, PIR, marital status, alcohol consumption, educational level, and race/ethnicity. WQS, weighted quantile sum.

**Figure 3:**
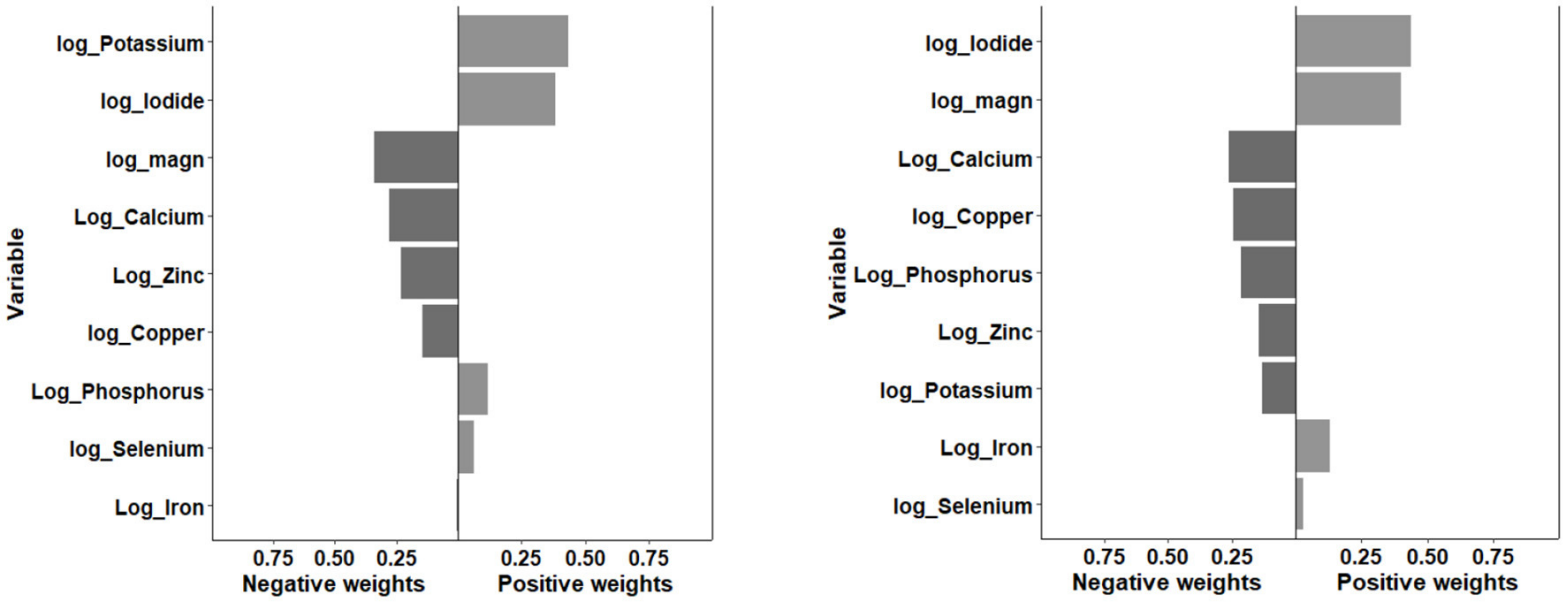
Qg-comp model regression index weights for hypothyroidism (figure left) and hyperthyroidism (figure right). The qg-comp models were adjusted by age, gender, energy intake, serum cotinine, BMI, PIR, marital status, alcohol consumption, educational level, and race/ethnicity. qg-comp, quantile g-computation.

Figures 2, 3 displayed the weight of each mineral for the overall effect on TD using WQS and qg-comp models. In WQS model, the mineral contributing most to the risk of hypothyroidism was zinc (mean weight = 0.323), followed by calcium (mean weight = 0.247), and copper (mean weight = 0.230); and the mineral contributing most to the risk of hyperthyroidism was copper (mean weight: 0.284), followed by calcium (mean weight: 0.271), and potassium (mean weight = 0.227). In qg-comp model, the mineral contributing to the risk of hypothyroidism were potassium and iodide for positive weight, and magnesium and calcium for negative weight; the mineral contributing to the risk of hyperthyroidism were iodide and magnesium for positive weight, and calcium and copper for negative weight.

**Figure 4:**
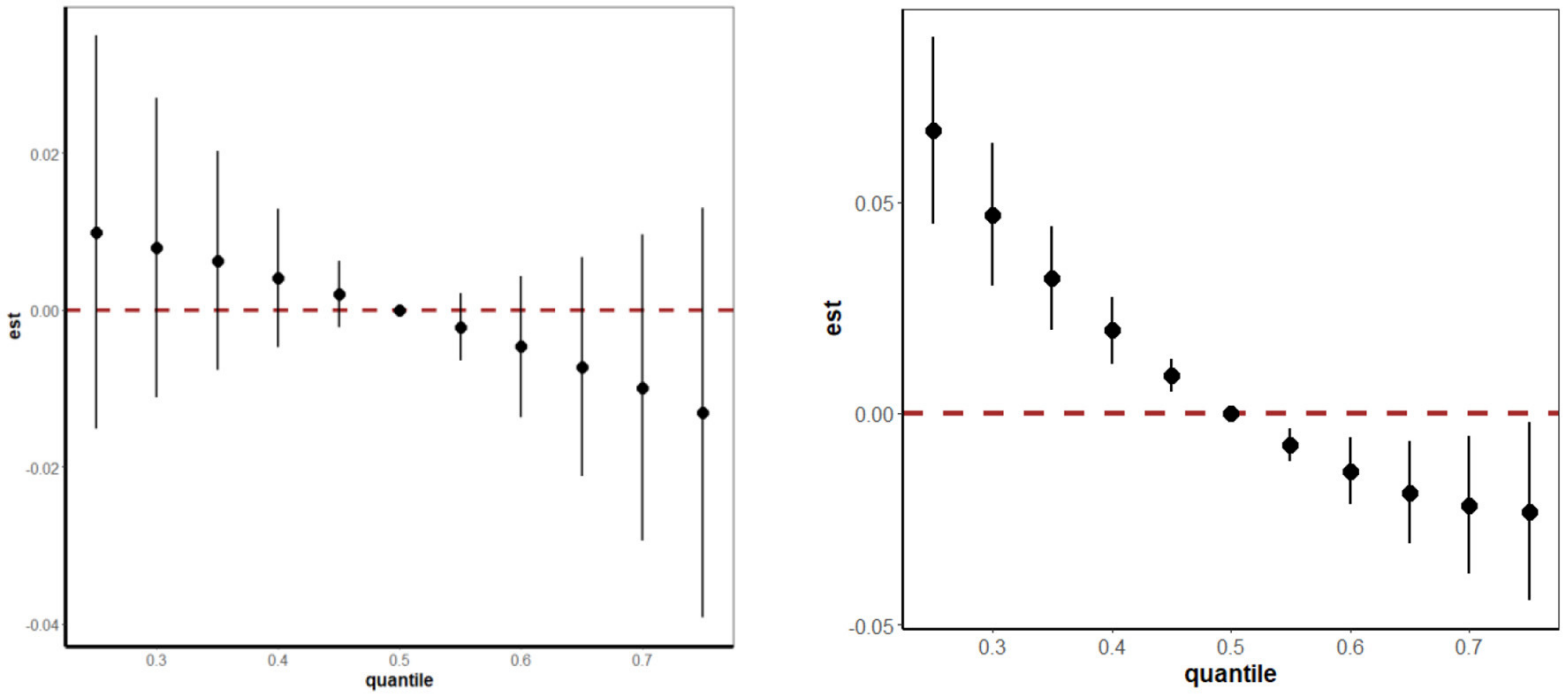
The overall effects of mixed minerals' intake on hypothyroidism (figure left) and hyperthyroidism (figure right) obtained by BKMR model. The BKMR models were adjusted by age, gender, energy intake, serum cotinine, BMI, PIR, marital status, alcohol consumption, educational level, and race/ethnicity. BKMR, bayesian kernel machine regression.

We further employed the BKMR model to estimate the overall effects of mixed minerals' intake on TD, which were exhibited in Figure 4. The overall effect showed that risk of hyperthyroidism decreased with the higher consumption of these nine minerals, with the difference being statistically significant. When the consumption of all minerals increased from the 30th percentile to 50th percentile, there was an observed approximate 5% decrease in the risk of hyperthyroidism. The decreasing trend in the risk of hypothyroidism was also observed with higher mineral consumption, although statistical significance was not reached.

Similar to the WQS and qg-comp models, the BKMR model also estimated the mineral-specific contribution to TD, referred to as the PIP, where a higher PIP indicates greater significance for TD. As shown in Table 4, the highest contributor to the risk of hypothyroidism was copper (PIP = 0.149), followed by magnesium (PIP = 0.087); the highest contributor to the risk of hyperthyroidism was magnesium (PIP = 0.648), followed by phosphorus (PIP = 0.335). Supplementary Figure S1 showed that the individual effect of each mineral on TD while holding other minerals at median values.

**Table 4:** Posterior inclusion probability (PIP) of the relationship between multiple minerals' intake and thyroid dysfunction.

**Minerals' intake**	**PIP (hypothyroidism)**	**PIP (hyperthyroidism)**
Calcium	0.037	0.001
Magnesium	0.087	0.648
Phosphorus	0.011	0.335
Iron	0.008	0.008
Zinc	0.028	0.011
Copper	0.149	0.024
Potassium	0.073	0.047
Selenium	0.072	0.022
Iodide	0.043	0.007

PIP, posterior inclusion probability. PIP suggests the relative significance of the impact on TD, with a higher PIP suggesting the greater significance to TD.

## 4 Discussion

The present study used data from the National Health and Nutrition Examination Survey (NHANES) to assess the effects of nine minerals' intake on the risk of TD in US adults aged 20 years and older using traditional logistic regression methods as well as three advanced machine learning methods. Diet and thyroid function are related closely. Poor diets such as vitamin and mineral deficiencies or excesses may increase the risk of TD (24–26). Maintaining normal thyroid function requires the co-regulation of several minerals (27). In this study, we found the negative joint effect of nine minerals on hyperthyroidism and hypothyroidism. Similar results were found in a Chinese study of 489 pregnant women, and they found that total concentrations of the six minerals were negatively correlated with TSH concentrations and positively correlated with FT3 and FT4 (28). Above results indicate that the maintenance of normal thyroid function necessitates the involvement of multiple minerals, rather than relying on a single mineral alone. Of them, the potential minerals contributing to the decreased risk of TD were calcium, zinc, and magnesium, while iodine may exert an opposite effect on TD. Meanwhile, we fail to found the significant results regarding the influence of selenium on TD.

In this study, calcium intake may be negatively associated with TD. In a Korean case-control study, high calcium intake was associated with a lower risk of thyroid cancer (25). To date, the epidemiological research examining the relationship between calcium intake and thyroid function remains limited. Calcium is known to be involved in numerous cellular activities in the body. In the thyroid gland, the expression of the thyrotropin receptor, the uptake of iodine by thyroid cells, and the dimerisation of thyroglobulin are dependent on the regulation of calcium (29). Moreover, calcium as important intracellular signaling molecules, are involved in various cellular processes, including hormone secretion. Research has found that calcium ions can directly act on thyroid cells through specific receptors or channels to regulate their functional activities (29). In clinical practice, patients with TD are advised to incorporate calcium-rich foods, such as dairy products, into their diet to support optimal thyroid function.

Zinc serves a critical role in human physiology, involving thyroid function. Lu et al. found that low dietary zinc intake was associated with the risk of hypothyroidism in a US population (24). This is consistent with the results of the present study. Moreover, some studies elucidated a substantial association between serum zinc levels and thyroid hormone concentrations (14, 30). A case-control study showed that higher serum zinc levels were associated with higher T3 and T4 levels found in hypothyroid populations, and lower serum zinc levels were observed in hypothyroid and hyperthyroid populations and compared to healthy populations (31). Hypothyroidism was also observed in rats fed a zinc-deficient diet, as evidenced by a significant reduction in the serum concentration of alkaline phosphatase, as well as a reduction in the concentration of hormonal indicators of thyroid function (32). As far as we know, zinc was identified as an important mineral with a protective effect on TD in the current study. A possible explanation is that zinc is also involved in the T4 to T3 conversion as part of the nuclear receptor proteins (33). In the thyroid gland, zinc is crucial for the function of thyroid peroxidase (TPO), an enzyme that is vital for producing thyroid hormones. TPO facilitates the iodination of thyroglobulin, which is a precursor protein for thyroid hormones, and also enables the coupling of iodotyrosine residues to generate T4 and T3 (34). Given the critical role of zinc in thyroid function and volume, we recommend that patients with TD incorporate zinc-rich foods such as oysters, fish, beans, nuts, red meat, whole grains, and dairy products into their diet.

Compared to other minerals, less research focused on magnesium. In this study, we found that magnesium intake was negatively associated with hyperthyroidism and hypothyroidism. Luo et al. found that serum magnesium concentrations were significantly lower in thyroiditis antibody-positive populations than in healthy populations, especially in women of reproductive age (35). In a recent meta-analysis, researchers also found lower blood magnesium levels in the thyroid cancer population (36). However, there are also contradictory results occurred in the previous literature. A large cross-sectional study that included 6,480 participants showed that higher serum magnesium and copper levels were positively associated with thyroid nodules (37). Magnesium contributes to thyroid hormone production as a stabilizing factor in nucleic acid structure as well as oxidative phosphorylation and ATP synthesis (38, 39). Animal studies have shown an independent correlation between hypomagnesemia and TD, especially hypothyroidism, highlighting the critical role of magnesium in iodine utilization by the thyroid gland and the conversion of T4 to its active form, T3 (40, 41). Therefore, maintaining a balanced magnesium intake through diet or supplements may be beneficial for supporting thyroid health.

Higher iodine intake has been consistently considered a strong risk factor for hyperthyroidism, and this notion was validated by both traditional and emerging machine learning models in the current study. Iodine is a crucial element for the normal functioning of the thyroid gland, directly involved in the synthesis and regulation of thyroid hormones (42). Compared to other minerals, the relationship between iodine and TD is clearer, and it has been widely reported that iodine excess and iodine deficiency impair thyroid function (43–45). Urinary iodine is one of the most common indicators used to evaluate iodine intake in humans (42). The current study showed that urinary iodine was positively associated with TD, which is consistent with previous studies. In addition, we observed that iodine accounted for a high positive weight in the joint exposure models. Experimental animal studies have shown that iodine overdose severely impairs the pituitary-thyroid axis in rodents (46, 47). Excessive iodine can lead to elevated TSH, which may be due to the fact that excessive iodine promotes the secretion of TRH from the hypothalamus, which causes elevated TSH, as well as affecting the activity of the important enzyme type II deiodinase, which inhibits the conversion of T4 to T3, and ultimately leads to elevated TSH levels (47–49). Based on the aforementioned findings, we conclude that iodine exerts a bidirectional influence on thyroid function. Therefore, it is imperative to maintain an appropriate level of iodine intake.

The association between selenium intake and thyroid function is inconclusive. The thyroid gland is the organ with the highest selenium content (50). Previous studies have suggested that low selenium diets were associated with goiter, hypothyroidism and thyroid nodules (51–53). Meanwhile, selenium supplementation has been used clinically, although a positive effect does not always occur (54). A previous large cross-sectional study included populations living in selenium-enriched areas and populations living in low-selenium areas, with both groups having similar dietary habits yet with significant differences in selenium intake due to natural influences (13, 55). The findings of that study implied that no significant association between serum selenium and hyperthyroidism was observed, however, lower serum selenium levels were associated with higher levels of clinical/subclinical hypothyroidism (13). Our study evaluated the association between selenium and TD, but no significant contribution of selenium was found in any of the models. The primary mechanisms linking selenium deficiency to thyroid disorders include the GPX enzyme superfamily that aids in antioxidant defenses, the deiodinase isoenzymes essential for activating and deactivating thyroid hormones, and the immune-related selenoproteins that regulate inflammatory responses and the interactions between immune cells and thyroid cells (56–58). These findings indicate a potential benefit of selenium supplementation for individuals with thyroid disease. However, larger and more comprehensive studies are necessary to conclusively establish these effects. Although selenium supplementation has been utilized clinically for treatment of thyroid-related diseases, the potential adverse effects brought from selenium supplementation must be carefully considered, particularly in individuals without selenium deficiency.

Multiple minerals are always consumed at the same time through food, and it is difficult to tell exactly which minerals play a role and whether there is a joint effect of these minerals. Few previous studies have been conducted on the relationship between minerals and thyroid diseases, and even fewer articles have examined the joint effects of minerals. Limited by traditional statistical methods is one of the reasons. Logistic regression incorporating a single mineral as the independent variable without considering the levels of other minerals made the results highly interpretable but with poor sensitivity, and only the effects of calcium, magnesium and potassium on hyperthyroidism were identified in that series of models. The joint effect between them and the relative importance of each mineral was not known. Fortunately, the emerging statistical methods of WQS, qg-comp and BKMR allow for mixed exposure analyses, which provide unique insights into the joint effects of minerals. They allowed a set of substances with strong correlations to enter the model simultaneously, and obtained empirical weights after millions of samples. In our WQS analysis, the negative joint effect of nine minerals on hyperthyroidism was statistically significant. The results of BKMR corroborate the results of WQS which is the significant negative joint effect of minerals on hyperthyroidism. However, WQS imposed the direction of mineral action, which may pose some challenges. Qg-comp did not make such constraints, and we still found a negative joint effect of minerals, in spite of the fact that there was no statistically significant difference. All results suggest that the maintenance of normal thyroid function requires the coordinated involvement of multiple minerals, rather than depending solely on a single mineral.

The current study is one of the first to assess the relationship between individual and mixed mineral intake and hyperthyroidism and hypothyroidism. The combined effect of mixed mineral intake on the risk of developing hyperthyroidism and hypothyroidism was assessed by combining traditional and machine learning methods and implied that the importance of the minerals differed in the joint effect. However, we do have to face the limitations of this study. Firstly, NHANES is a cross-sectional study, which does not support the determination of a causal relationship between minerals' intake and hyperthyroidism and hypothyroidism. Second, we included only 9 common minerals, which is indicative to some extent but not exhaustive, and perhaps there are other minerals in trace amounts but with important implications that we did not include in the study. Third, the use of 24-h dietary recalls to assess dietary minerals' intake is informative, but may not be a representative assessment of long-term exposure. The current inclusion of data from the 4 NHANES cycles does not provide complete data on serum mineral levels. Nevertheless, combining serum mineral levels with dietary mineral intake may provide a more complete explanation. Last, besides dietary mineral consumption, there are any possible comorbidity in TD patients that might be caused by the excretion of dietary trace elements from the body, which may influence the results in this study.

## 5 Conclusion

In conclusion, the current study explored the association of nine mineral intakes with TD by employing traditional regression models and three advanced machine learning models. The machine learning models reported the negative joint effect of the nine mineral intakes on TD and implied a different weighting of the effect of each mineral in the joint effect. The intake levels of various minerals exhibit varying degrees of association with TD. Maintaining a balanced intake of multiple minerals may be advantageous in the prevention and treatment of TD.

## Data Availability

Publicly available datasets were analyzed in this study. This data can be found at: https://www.cdc.gov/nchs/nhanes/.
