# Supplementary material for: Machine learning-based exploration of the associations between multiple minerals' intake and thyroid dysfunction: data from the National Health and Nutrition Examination Survey
Source: Front Nutr. 2025 Mar 26;12:1522232. doi: 10.3389/fnut.2025.1522232 (PMC11978632; doi:10.3389/fnut.2025.1522232)
Supplement: Supplementary file 1 [file Image_1.pdf]

Machine Learning-based Exploration of the associations between multiple minerals' intake and thyroid dysfunction: Data from the National Health and Nutrition Examination Survey

Shaojie Liu[1, 2, a], Weibin Huang[3, a], Yaming Lin[1, a], Yifei Wang[2], Hongjin Li[4], Xiaojuan Chen[1], Yijia Zou[1], Bo Chen[2], Baochang He[5, *], Zhiping Yang[1, *], Jing Fan[2, *]

[1] The First Affiliated Hospital of Xiamen University, School of Medicine, Xiamen University, Xiamen 361003, China
[2] Key Laboratory of Public Health Safety of Ministry of Education, School of Public Health, Fudan University, Shanghai 200032, China
[3] Department of Neurology, The First Affiliated Hospital of Fujian Medical University, Fuzhou 350005, China
[4] Institute for Infectious Disease Control and Prevention, Fujian Provincial Center for Disease Control and Prevention, Fuzhou 350012, China
[5] School of Public Health, Fujian Medical University, Fuzhou 350122, China
[a] These authors contributed equally to this work
[*] Correspondence: Jing Fan, Email: jfan21@m.fudan.edu.cn; Zhiping Yang, Email: zhipingyang2020@163.com; Baochang He, Email: hbc517@163.com

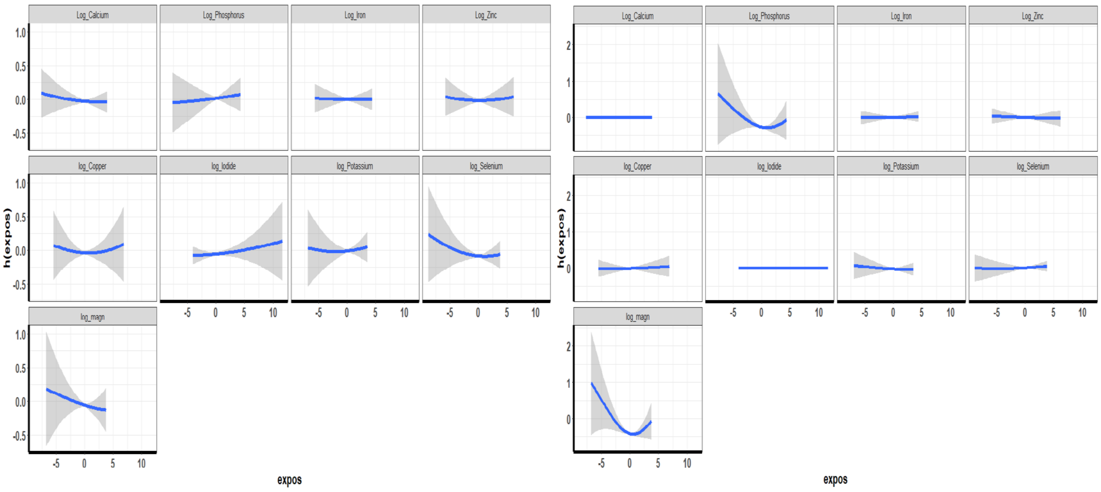

Figure S1. Univariate exposure-response functions and 95% confidence interval for each mineral on the effect of hypothyroidism(left) and hyperthyroidism (right), with other minerals fixed at the median.
